# Characterization of newly established Pralatrexate-resistant cell lines and the mechanisms of resistance

**DOI:** 10.1186/s12885-021-08607-9

**Published:** 2021-07-31

**Authors:** Kana Oiwa, Naoko Hosono, Rie Nishi, Luigi Scotto, Owen A. O’Connor, Takahiro Yamauchi

**Affiliations:** 1 Department of Hematology and Oncology, Faculty of Medical Sciences, University of Fukui, 23-3 Matsuokashimoaizuki, Eiheiji-cho, Yoshida-gun, Fukui, 910-1193 Japan; 2 The Center of Lymphoid Malignancy, Columbia University Medical Center, College of Physicians and Surgeons, 630 West 168th St, New York, NY 10032 USA; 3 Department of Medicine, Division of Hematology and Oncology, University of Virginia, 1215 Lee Street, Charlottesville, VA 22903 USA

**Keywords:** Pralatrexate, Resistance, DNA-methyltransferase 3β, Decitabine

## Abstract

**Background:**

Pralatrexate (PDX) is a novel antifolate approved for the treatment of patients with relapsed/refractory peripheral T-cell lymphoma, but some patients exhibit intrinsic resistance or develop acquired resistance. Here, we evaluated the mechanisms underlying acquired resistance to PDX and explored potential therapeutic strategies to overcome PDX resistance.

**Methods:**

To investigate PDX resistance, we established two PDX-resistant T-lymphoblastic leukemia cell lines (CEM and MOLT4) through continuous exposure to increasing doses of PDX. The resistance mechanisms were evaluated by measuring PDX uptake, apoptosis induction and folate metabolism-related protein expression. We also applied gene expression analysis and methylation profiling to identify the mechanisms of resistance. We then explored rational drug combinations using a spheroid (3D)-culture assay.

**Results:**

Compared with their parental cells, PDX-resistant cells exhibited a 30-fold increase in half-maximal inhibitory concentration values. Induction of apoptosis by PDX was significantly decreased in both PDX-resistant cell lines. Intracellular uptake of [^14^C]-PDX decreased in PDX-resistant CEM cells but not in PDX-resistant MOLT4 cells. There was no significant change in expression of dihydrofolate reductase (DHFR) or folylpolyglutamate synthetase (FPGS). Gene expression array analysis revealed that DNA-methyltransferase 3β (DNMT3B) expression was significantly elevated in both cell lines. Gene set enrichment analysis revealed that adipogenesis and mTORC1 signaling pathways were commonly upregulated in both resistant cell lines. Moreover, CpG island hypermethylation was observed in both PDX resistant cells lines. In the 3D-culture assay, decitabine (DAC) plus PDX showed synergistic effects in PDX-resistant cell lines compared with parental lines.

**Conclusions:**

The resistance mechanisms of PDX were associated with reduced cellular uptake of PDX and/or overexpression of DNMT3B. Epigenetic alterations were also considered to play a role in the resistance mechanism. The combination of DAC and PDX exhibited synergistic activity, and thus, this approach might improve the clinical efficacy of PDX.

**Supplementary Information:**

The online version contains supplementary material available at 10.1186/s12885-021-08607-9.

## Background

Peripheral T-cell lymphomas (PTCLs) are a heterogeneous group of mature T-cell and natural killer-cell neoplasms accounting for approximately 5–15% of non-Hodgkin’s lymphomas [1]. Owing to intrinsic chemotherapy resistance, the prognosis of patients with PTCL is extremely poor. The median time of overall survival of patients after first relapse is estimated to be only 5.5 months [2, 3]. Conventional chemotherapy regimens have shown limited activity in the second-line setting and beyond, thereby creating a need for new drugs that are selectively active against T-cell malignancies.

Recently, pralatrexate (10-propargyl 10-deazaminopterin, PDX) has been approved for the treatment of PTCL in the USA, Japan, and other countries. PDX is a folate analog designed to have a high affinity for reduced folate carrier (RFC1), dihydrofolate reductase (DHFR), and folylpolyglutamate synthetase (FPGS), resulting in increased cytotoxic activity compared with methotrexate (MTX) [4, 5]. PDX is transported into cancer cells via RFC1 and undergoes polyglutamylation by FPGS, leading to subsequent inhibition of DHFR and termination of DNA synthesis. Although this mechanism of DHFR inhibition is common for antifolates, PDX may also have other effects in PTCLs.

MTX is a well-established antifolate that blocks the action of DHFR, and has been used to treat various types of non-Hodgkin’s lymphomas, including primary central nervous system lymphoma and Burkitt’s lymphoma. However, its clinical efficacy in PTCL is limited. PDX has been studied across a variety of non-Hodgkin’s lymphomas [6–8] and is most efficacious in patients with PTCL, leading to its accelerated approval by the US Food and Drug Administration for patients with relapsed/refractory PTCL. A recent case match control analysis confirmed that there was a survival advantage for patients enrolled in the Pralatrexate in Patients with Relapsed or Refractory Peripheral T-Cell Lymphoma (PROPEL) study who received PDX compared with a well-matched population of patients receiving the standard of care. These data suggested an overall survival advantage in the PDX-treated population (14.5 months) versus the control population (4 months) [7]. Because of the apparent T-cell selective activity of PDX, this drug has been studied in combination with a host of agents, including romidepsin and 5-azacytidine.

Despite this promising activity, some patients can acquire resistance to PDX over time. Identifying the underlying mechanisms of drug resistance could lead to rational strategies to prevent or overcome these mechanisms of resistance, thereby improving clinical efficacy. Accordingly, in this study, we newly established two PDX-resistant T-lymphoblastic leukemia cell lines designated CCRF-CEM (CEM) and MOLT-4 (MOLT4) to explore these mechanisms of drug resistance. These cell lines were then used to develop and evaluate complementary drug combinations as a means to overcome acquired resistance to PDX.

## Methods

### Cell lines and culture

The human acute T-lymphoblastic leukemia cell lines CCRF-CEM (CEM) and MOLT4 were purchased from JCRB Cell Bank (Osaka, Japan, CEM:JCRB0033, MOLT-4:JCRB9031) in 2015. The cells were cultured in RPMI-1640 medium supplemented with 10% fetal bovine serum at 37 °C under 5% CO_2_ in a humidified atmosphere.

Spheroid (3D) cell culture was performed with Col-Tgel (101 Bio, CA) following manufacture protocol. In brief, 2 × 10^6^ cells/100 μL were seeded in soft medium into 48-well plates. The cells were placed in a standard CO_2_ cell culture incubator (37 °C, 5% CO_2_).

### Reagents

PDX and forodesine (FDS) were kindly provided by Mundipharma K.K. (Tokyo, Japan). FDS is a novel inhibitor of purine nucleoside phosphorylase and was approved for the treatment of relapsed/refractory PTCL in Japan [9]. MTX, cytarabine (AraC), bortezomib (BOR), decitabine (DAC), deoxyguanosine (dGuo), and panobinostat (LBH589) were purchased from Sigma-Aldrich Japan (Tokyo, Japan). PDX, DAC, and dGuo were dissolved in dimethyl sulfoxide; MTX and BOR were dissolved in phosphate-buffered saline; and FDS and AraC were dissolved in pure water.

### Establishment of resistant cell lines

CEM and MOLT4 cells were initially incubated with 0.01 nM PDX, and the concentration of PDX was then gradually increased by 0.01–0.02 nM over a period of 10 months. The initial concentration was 1/100 the concentration required to inhibit 50% growth of the cells (IC_50_). After acquisition of PDX-resistant cells, single-cell cloning was performed by a limiting dilution method according to previous reports [10–13]. In brief, the culture medium containing PDX-resistant cells (1.0 × 10^5^ cells/mL) was diluted 10,000-fold, and cells were dispensed into 96-well plates at a density below one cell per well. After culturing the cells for approximately 3 weeks, a single clone was obtained from each well. Eleven PDX-resistant CEM (CEM/P) single-cell clones and 19 PDX-resistant MOLT4 (MOLT4/P) single-cell clones were obtained, and clones that showed the most vigorous proliferation were utilized for further experiments.

### Growth inhibition assay

The IC_50_ value of each drug was calculated from an analysis of growth inhibition. To evaluate the proliferative activity of each cell line, 2,3-bis-(2-methoxy-4-nitro-5-sulfophenyl)-2 h-tetrazolium-5-carboxanilide (XTT) assays were performed according to the manufacturer’s instructions (Roche, Indianapolis, IN, USA) [10, 14–18]. Spheroid (3D) cell viability of Col-Tgel encapsulated cells was also assessed using XTT assays. The combination index (CI) of PDX and DAC was calculated using COMPUSYN software (http://www.combosyn.com).

### Quantification of apoptotic cell death

The percentage of apoptotic cells was assessed by flow cytometry using an Annexin V-FLUOS Staining kit (Roche). The cells were treated with PDX or FDS for 48 h, washed, and stained with propidium iodide, annexin V-FITC, or both, according to the manufacturer’s instructions. The cells were then analyzed using a FACSCantII flow cytometer (BD Bioscience, Franklin Lakes, NJ, USA). Annexin V-positive cells were considered to be apoptotic.

### Gene expression analysis by reverse transcription-quantitative polymerase chain reaction (RT-qPCR) and microarray

RT-qPCR was performed by a two-step reaction. RNA from each cell line was prepared using RNeasy Mini Kit (Qiagen, Hilden, Germany). cDNA was then synthesized using a PrimeScript RT reagent kit (Takara, Shiga, Japan), and qPCR was conducted using a TaqMan Fast Advanced Master Mix kit (Applied Biosystems, Waltham, MA, USA). Primers for *RFC1* (Hs01099126), DNA methyltransferase 3β (*DNMT3B*; Hs00171876), and glyceraldehyde 3-phosphate dehydrogenase (*GAPDH;* Hs2786624) were purchased from Applied Biosystems. The microarray analysis was performed by Clariom S Arrays (Thermo Fisher Scientific K. K, Tokyo, Japan) using 10 ng total RNA extracted from parental and PDX-resistant cells.

#### DNA methylation analysis

Genomic DNA extracted from parental and PDX-resistant cell lines. DNA was was treated with sodium bisulphite using the EZ DNA methylation Gold Kit (Zymo Reserch,CA, USA) according to manufacturer’s instructions. DNA methylation was quantified using the Illumina Infinium HumanMethylation450 (HM450) and HumanMethylationEPIC (EPIC) BeadChip (Illumina, CA, USA) run on an Illumina iScan System (Illumina, CA, USA) using the manufacturer’s standard protocol.

### Western blot analysis

Western blotting analysis was performed using standard protocols as published elsewhere [10, 14]. Briefly, protein lysates were extracted from the cells (1 × 10^7^ cells) using a Qproteome Mammalian Protein Prep Kit (Qiagen), and the lysates were applied to 7.5% sodium dodecyl sulfate-polyacrylamide gel electrophoresis gels for separation. Proteins were then transferred onto Immobilon-P membranes (Millipore, Billerica, MA, USA). The membranes were probed with primary and secondary antibodies using standard techniques. Anti-FPGS (cat. no. ab184564; Abcam, Cambridge, UK), anti-DHFR (cat. no. 872442; R&D Systems, Minneapolis, MN, USA), anti-caspase 3 (cat. no. 9665; Cell Signaling Technology, Massachusetts, USA), anti-PARP (cat. no. 9542; Cell Signaling Technology), anti-cleaved PARP (cat. no. 9541; Cell Signaling Technology), and anti-β-actin (cat. no. A2066l Sigma-Aldrich Japan) antibodies were used as primary antibodies, and anti-rabbit polyclonal antibodies (cat. no. 7074; Cell Signaling Technology, Tokyo, Japan) were used as secondary antibodies. Protein detection and quantification were performed using Amersham ECL Prime Western Blotting Detection Reagent and an ImageQuant LAS4000mini system (GE Healthcare Life Sciences, Little Chalfont, UK).

### Cellular uptake of [^14^C]-PDX

Cellular uptake of PDX was calculated by a radioisotope assay. The cells (5 × 10^6^) were incubated with 1 nM [^14^C]-PDX for 0, 5, 10, 20, or 30 min, and cell pellets were dissolved using SOLUENE-350 and Clear-sol l (Nacalai Tesque, Kyoto, Japan). Radioactivity was measured using a liquid scintillation counter.

### Statistical analyses

Statistical analyses and graph generation were performed using GraphPad Prism (version 6.0. GraphPad Software, San Diego, CA, USA).

## Results

### Establishment of two PDX-resistant cell lines

**Fig. 1:**
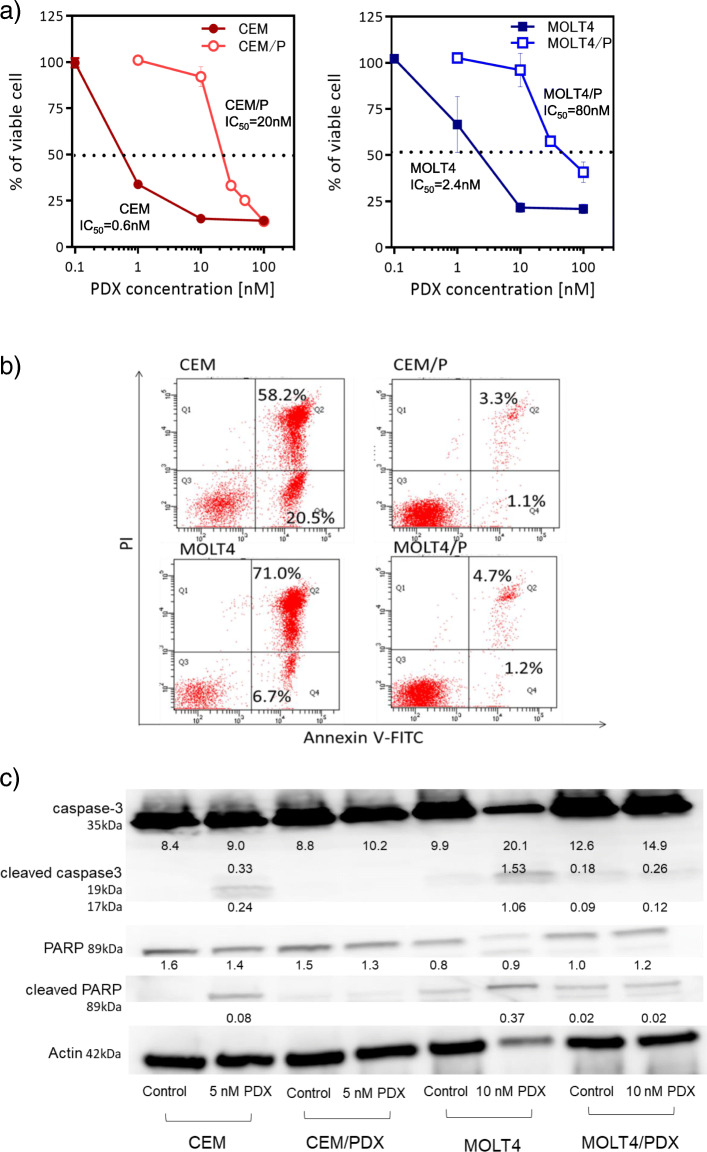
Establishment of PDX resistance. **a**) Dose response growth inhibition curves for PDX. Growth inhibition curve relative to untreated control of T-ALL cell lines CEM and MOLT4. Cells were treated with various concentration of PDX for 72 h and cell viability was measured using the XTT assay. Individual IC_50_ values were determined from curve fitting. **b**) Induction of apoptosis by PDX. After 72 h of PDX treatment at the indicated concentration (CEM and CEM/P cells: 5 nM, MOLT4 and MOLT4/P cells: 10 nM), cells were stained with Annexin V-FITC and PI and analyzed by flow cytometry. The percentage of cells in each group within the gated areas is indicated; the upper right panel represents cells undergoing late apoptosis, and the lower right panel represents cells undergoing early apoptosis. **c**) PDX induced caspase activation. CEM and MOLT4 cells were treated with PDX (CEM and CEM/P cells: 5 nM, MOLT4 and MOLT4/P cells: 10 nM) for 48 h. Western blots analysis of caspase-3 and PARP cleavage were performed to characterize the apoptotic response. Beta-actin was used to normalized proteins contents and band intensity values are shown below the corresponding band. Results are representative of three independent experiments. PDX, pralatrexate. CEM/P, PDX-resistance CEM cell. MOLT4/P, PDX-resistance MOLT4 cell

To generate PDX-resistant cell lines, the human acute T-lymphoblastic leukemia cell lines CEM and MOLT4 were exposed to gradually increasing PDX concentrations for 10 months. The half-maximal inhibitory concentration IC_50_ values for the PDX-resistant cell lines (CEM/P and MOLT4/P) were 20 nM and 80 nM, respectively. In comparison with the IC_50_ values of the parental cells (CEM: 0.6 nM, MOLT4: 2.4 nM), those of the PDX-resistant cell lines were increased by approximately 33-fold (Fig. 1a). The doubling times of PDX-resistant cells were similar to those of their parental counterparts (Supplementary Data 1), and the degree of resistance in these cells did not change for 6 months despite culturing the cells in medium without PDX.

To assess PDX-induced cytotoxicity, we evaluated the induction of apoptosis using flow cytometry. After 48 h of treatment with PDX at the IC_75_ (5 nM for CEM cells, 10 nM for MOLT4 cells), induction of apoptosis was observed in 78.7% of CEM cells and 77.7% of MOLT4 cells, whereas only 4.4% of CEM/P cells and 5.9% of MOLT4/P cells were apoptotic at the same concentrations (Fig. 1b). To confirm that PARP1 cleavage occurred by PDX treatment, total and cleaved PARP1 and caspase 3 were assessed in both parental and PDX-resistant cell lines (Fig. 1c). Cleaved PARP1 and caspase 3 were observed in both parental cell lines but not in the PDX-resistant cell lines, suggesting that no effective induction of apoptosis occurred in PDX-resistant cells.

### Intracellular uptake of PDX

**Fig. 2:**
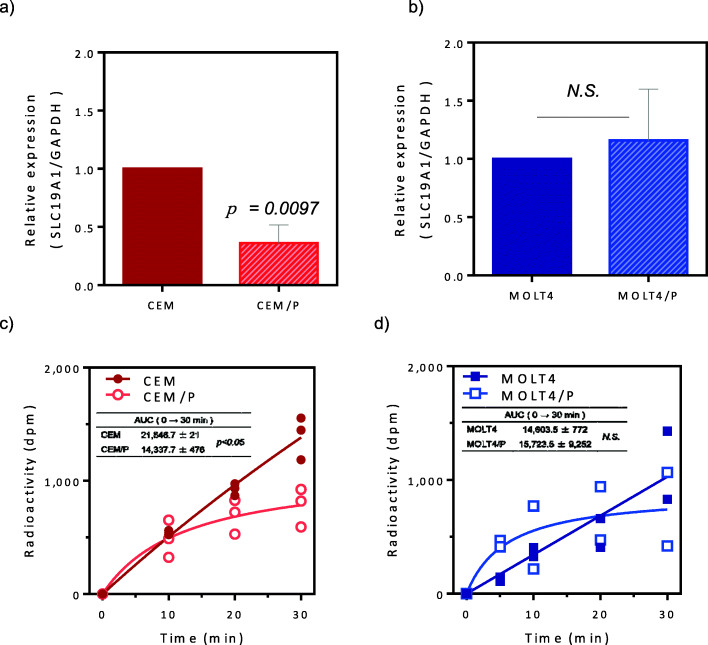
Expression levels of RFC1 and PDX uptake. **a, b**) Quantitative analysis of RFC1(SLC19A1) mRNA by reverse transcription-quantitative polymerase chain reaction. Fold change compared with the expression levels in corresponding control cells are presented. Each data point represents the mean ± standard deviation of three independent experiments. Statistical analysis was performed using two-tailed pares Student’s t-tests. **c, d**) Cellular uptake of [^14^C]-PDX. The cells were incubated with 1 nM [^14^C]-PDX for designated time periods (0, 10, 20, 30 min) and centrifuged to terminate the reaction. The cell pellet was then dissolved and evaluated for radioactivity using a liquid scintillation counter. Mean values and maximal variations from three measurements are shown. Inserted table, the amount of [^14^C]-PDX uptake was shown as a median value of the area under the curve. Statistical analysis was performed using two-tailed pares Student’s t-tests

We next focused on the intracellular uptake of PDX because acquired mechanisms of resistance to MTX have been attributed to decreased expression or inactivating mutations of RFC1, resulting in decreased MTX internalization [19–22]. RT-qPCR analysis showed that the mRNA expression level of *RFC1* in CEM/P cells was significantly decreased compared with that in parental CEM cells (Fig. 2a). Accordingly, intracellular uptake of [^14^C]-PDX was significantly decreased in CEM/P cells. The area under the curve values were 21,646.7 dpm·min in CEM cells and 14,337.7 dpm·min in CEM/P cells (*p* = 0.0097; Fig. 2c). There were no significant differences in *RFC1* expression or intracellular uptake of [^14^C]-PDX between MOLT4 and MOLT4/P cells (Fig. 2b and d). Sequencing analysis of exons 2 and 3 in *RFC1,* which contain the MTX efflux site involved in antifolate resistance [21, 22], showed no acquired somatic mutations in PDX-resistant cells (Supplementary Data 2).

### Expression levels of FPGS and DHFR

**Fig. 3:**
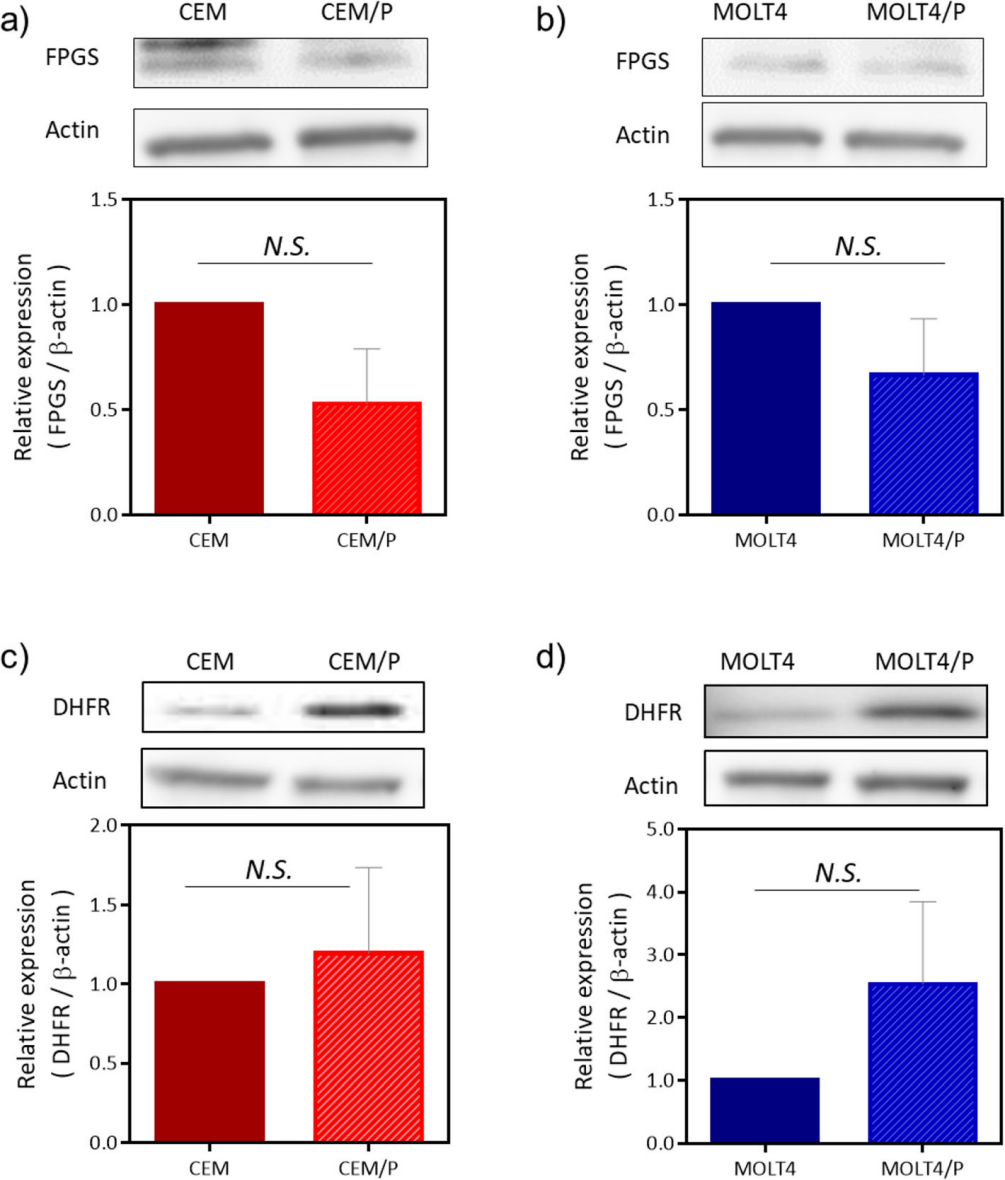
Expression levels of FPGS and DHFR. Western blot analysis of FPGS (a,b) and DHFR (c,d) from parental and PDX-resistant cells lysates. Beta-actin was used as loading control. The bar graph indicated the fold changes of protein expression using by quantitative immunoblotting. Blots are representative of three independent experiments. N.S., not significant

The resistance mechanisms associated with intracellular folate metabolism are thought to be related to deficiencies in FPGS activity [17, 18, 23] and/or increased DHFR expression [24–27]. FPGS catalyzes the formation of polyglutamate chains, yielding active PDX polyglutamates. Therefore, we examined the expression levels of FPGS protein in PDX-resistant cells and parental cells and observed no significant differences in either pair of cell lines (Fig. 3a, b). Furthermore, the protein expression level of DHFR, a key enzyme involved in intracellular folate metabolism and the induction of intrinsic resistance to MTX, was not significantly different in either pair of cell lines (Fig. 3c, d).

### Patterns of cross-resistance to other anticancer agents

To assess acquired drug resistance in PDX-resistant cells, the growth inhibitory effects of other anticancer drugs were compared between parental cells and PDX-resistant cells, and the IC_50_ values and relative degree of resistance were determined (Table 1). As expected, CEM/P and MOLT4/P cells were 2.1- and 3.0-times more resistant to MTX, respectively. The sensitivity of the cells to nucleoside analogs was more prominent in PDX-resistant cells; the relative degree of resistance to forodesine (FDS) was 0.4 in CEM/P cells and that to cytarabine (AraC) was 0.1 in MOLT4/P cells. On the other hand, CEM/P cells showed moderate resistance to bortezomib (BOR), and the relative degree of resistance was 1.8. No cross-resistance to LBH589 was observed in PDX-resistant cell lines.

**Table 1:** IC_50_ values of PDX and other agents

	IC_50_ (nM)					
	CEM	CEM/P	[ratio]	MOLT4	MOLT4/P	[ratio]
PDX	0.6	20	33	2.4	80	33
MTX	8.5	18	2.1	40	120	3.0
FDS^a^	4.2	1.6	0.4	8.0	7.0	0.9
AraC	30	35	1.2	24	2.0	0.1
BOR	3.7	6.5	1.8	5.9	6.5	1.1
LBH589	5.5	5.0	0.9	5.5	6.2	1.1
DAC	6.5 × 10^3^	2.3 × 10^3^	0.4	3.7 × 10^3^	750	0.2

*IC*_*50*_ 50% growth inhibitory concentration, *PDX* pralatrexate, *MTX* methotrexate, *FDS* forodesine, *AraC* cytarabine, *BOR* bortezomib, *LBH589* panobinostat, *DAC* 5-aza-2′-deoxycytidine

^a^In assays using FDS, cells were incubated with deoxyguanosine (10 μM) because the cytotoxicity of FDS requires the presence of deoxyguanosine in vitro

### Gene expression and methylation analysis

**Fig. 4:**
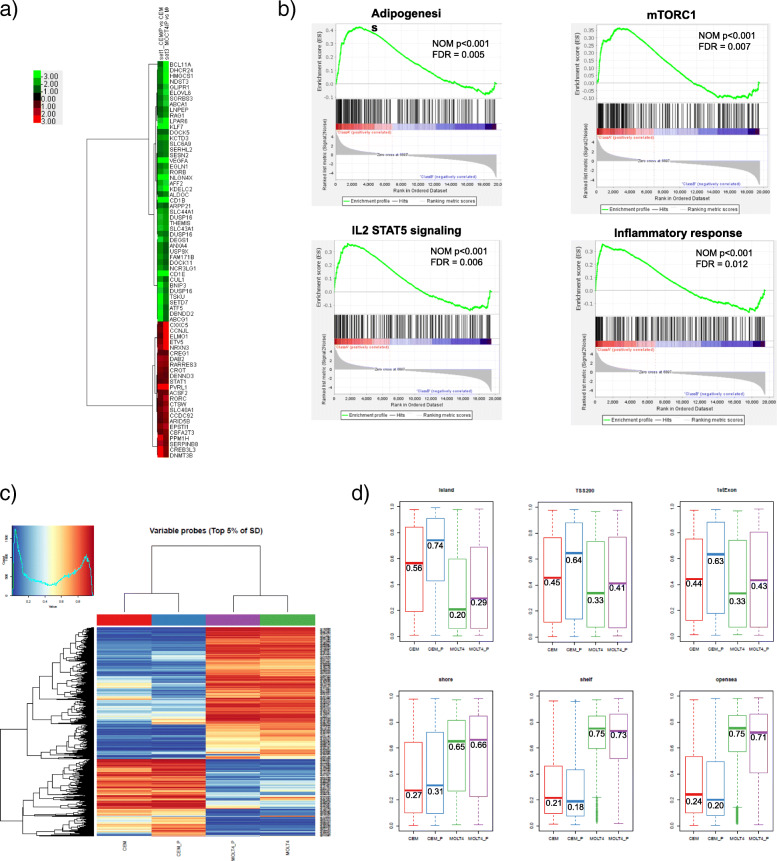
Microarray and methylation analysis. **a**) Microarray analysis from parental and PDX-resistant cells. Hierarchical cluster analysis of gene expression profiles indicated the 72 genes with commonly expression changes (more than 2-fold or less than 0.5-fold) between the PDX-resistance cell lines. The scale shows the level of expression, where red indicates increased gene expression, green indicates decreased expression, and the intensity of color correlated to the magnitude change. **b**) Gene set enrichment analysis. Significantly enriched gene signatures in GSEA analysis with microarray analysis from parental and PDX-resistant cells using the hallmark gene set. NOM; nominal, FDR; false discovery rate. **c**) DNA methylation heatmap showing unsupervised clustering of the top 5% most variable genes among PDX-resistant cell. **d**) The boxplot indicates the distribution of mean differences of beta values between of the CpG islands, shore, open sea, TSS 200 and 1st exon

To identify the potential mechanisms underlying PDX resistance, we performed mRNA expression profiling of PDX-resistant and parental cells using microarray analysis. We identified 4227 and 4034 significantly deregulated transcripts (more than 2-fold or less than 0.5-fold) in CEM/P and MOLT4/P cells, respectively. Most of the differentially expressed genes were unique to each cell line, and only 72 genes were commonly up- or downregulated between the PDX-resistance cell lines (Fig. 4a). Among them, the DNA-methyltransferase 3β (*DNMT3B)* expression level was significantly elevated in both CEM/P and MOLT4/P cells versus their parental cells. There were no significant differences in the expression levels of folate metabolism pathway components such as *FPGS, DHFR*, and thymidylate synthase (*TS*) in either PDX-resistant cell line (Supplementary Data. 3). Gene set enrichment analysis (GSEA) was performed for the PDX-resistant cell line comparison using hallmark gene sets collections in the Molecular Signatures Database (MSigDB). Four gene sets, namely the adipogenesis, mechanistic target of rapamycin complex1 (mTORC1) signaling, IL2/STAT5 signaling and inflammatory response gene sets, were commonly correlated with the PDX-resistance (Fig. 4b). The GSEA results of individual cell lines are shown in Supplementary Data 4.

Next, we focused on epigenetic changes in PDX-resistant cells because the *DNMT3B* expression level was significantly elevated in both resistant cell lines. DNA methylation arrays were applied, and the heatmap of DNA methylation levels of the top 5% of the data (2 standard deviations) is shown in Fig. 4c. As expected, the methylation status of CpG islands were increased in both PDX-resistant cell lines versus their parental cell lines (Fig. 4d). Furthermore, methylation sites from TSS 200 and 1st exon regions were also enriched in methylation signature of PDX resistance. There were no significant changes in the methylation status of other regions, such as the shore, shelf or open sea. We explored DNA promoter methylation as a potential mechanism of expression change. For genes with reduced expression in both PDX-resistant cells, *KDELC2* gene, a glucosyltransferase protein, had increased methylation in CpG islands. The *CUL1* and *EGLN1* genes had also increased methylation in the shore. On the other hand, for genes with increased expression in both PDX-resistant cells, *CBFA2T3*, a transcription corepressor, had decreased methylation in CpG island. There were no significant changes in methylation for specific genes involved in folic acid metabolism, such as *RFC1*, *FPGS*, *DHFR* and *TYMS*, or genes involved in DNA methyltransferase (*DNMT1*, *DNMT3a* and *DNMT3b*). We also examined PDX sensitivity in DNMT3B knockdown cells, and the IC_50_ values were 30 and 18 nM in CEM/P-mock and CEM/P-shDNMT3B cells, respectively, suggesting limited recovery of PDX sensitivity (Supplemental Data. 5).

### Enhancement of the cytotoxicity of PDX with decitabine (DAC) in PDX-resistant cells

**Fig. 5:**
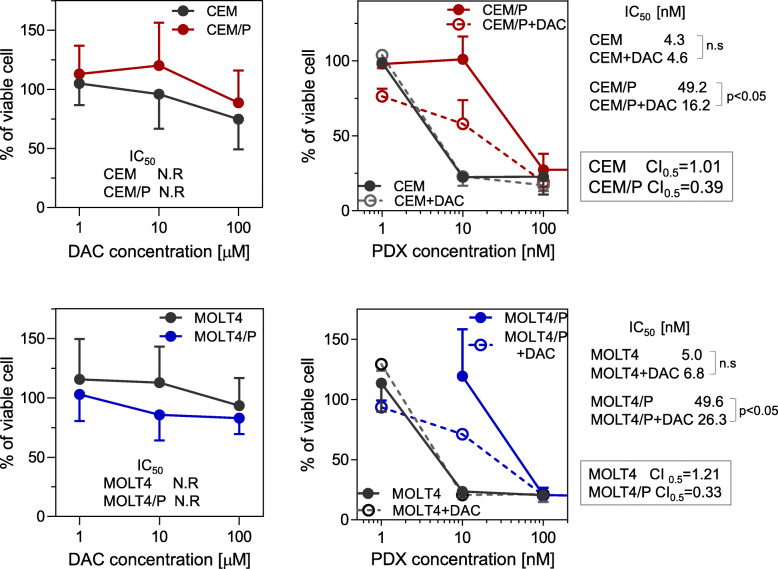
Combination of PDX with DAC in 3D-culture. Parental or PDX-resistant cells were incubated with various concentrations of PDX and DAC (CEM and CEM/P cells:100 μM, MOLT4 and MOLT4/P cells:10 μM) for 72 h and cell viability was measured using the XTT assay. Individual IC_50_ values were determined from curve fitting. The combination index (CI) of PDX and DAC was calculated using COMPUSYN software (http://www.combosyn.com). CI defines synergism (CI < 1), additive effect (CI = 1) and antagonism (CI > 1). Statistical analysis was performed using two-tailed paired Student’s t-tests. N.R: not reached

Finally, we evaluated the advantage of combining PDX with DAC, an inhibitor of DNMT, which could pharmacologically counterbalance the high expression of DNMT3B, using a 3D culture system. Treatment with DAC significantly inhibited the cell growth in combination with PDX. Synergistic effects (CI = 0.33–0.39) were observed at the IC_50_ in both PDX-resistant cell lines (Fig. 5). This synergism was specific to PDX-resistant cells, as the combination of PDX and DAC in parental cells only showed an additive or antagonistic effect (CI = 1.00–1.20). These results suggested that epigenetic agents may overcome cellular resistance to PDX.

## Discussion

Using classical methods to produce drug-resistant cell lines, we generated two cell lines that showed approximately 30-fold greater resistance to PDX than their parental counterparts. The characteristics of the PDX-resistant cells were as follows: (i) decreased internalization of PDX in CEM/P cells owing to reduced RFC expression; (ii) increased expression of DNMT3B; (iii) hypermethylation of CpG islands; and (iv) synergism with the hypomethylating agent DAC.

The potential mechanisms of acquired PDX resistance have gradually been uncovered in recent years [24, 28, 29], and appear to be similar to the resistance mechanism of MTX which have been extensively studied [30, 31]. The most frequent mechanism mediating antifolate resistance is related to the impairment of antifolate uptake, typically due to reduced expression of RFC1 and/or inactivating mutations in RFC1 [21, 22, 28, 32]. Increased antifolate efflux owing to overexpression of multidrug resistance efflux transporters has also been reported [30, 31]. Mechanisms related to defective polyglutamylation have been shown to be attributed to decreased FPGS expression, inactivating mutations in FPGS, and increased expression of gamma glutamyl hydrolase (GGH) [30, 31]. In addition, amplification of *DHFR* and/or *TS* caused by overexpression has been reported, and mutations in these genes can decrease their affinity for antifolates, resulting in antifolate resistance [26, 30, 31]. Our results indicated that impaired internalization of PDX owing to decreased expression of RFC1 may be one of the mechanisms of PDX resistance in CEM/P cells. In contrast, there were no obvious changes in PDX uptake or folate metabolism-related proteins in MOLT4/P cells, suggesting that other mechanisms were involved.

As shown in Fig. 4, the expression level of DNMT3B was increased in both PDX-resistant cell lines. Furthermore, gene set enrichment analysis revealed that PDX resistance was related to adipogenesis, mTORC1, the inflammatory response and IL-2/STAT5 signaling pathway. Overexpression of DNMT3B is related to drug resistance [33, 34] and the mTORC1 pathway is known to play an important role in the pathogenesis of lymphoma and various type of cancer [35], suggesting that these mechanisms may contribute to PDX resistance. In addition, DNMT3B plays an important role in the methylation of CpG islands, and as expected, PDX-resistant cells also had increased methylation of the CpG locus. Knockdown of DNMT3B in CEM/P cells resulted in only partial restoration of PDX resistance, and no specific hypermethylation gene that correlates with PDX resistance was detected, but it is speculated that hypermethylation might be associated with PDX resistance.

The combination with DAC resulted in high susceptibility to PDX in our resistant cells (Fig. 5), but did not show a synergistic effect in parental cells. Recently, several studies have explored potentially synergistic combinations with PDX [15, 16, 29, 36–39]. For example, T-cell lymphomas are driven by some epigenetic defects [39], and sensitivity to epigenetic therapies such as histone deacetylase (HDAC) inhibitors (e.g., romidepsin) or DNMT inhibitors [38] has been noted. The combination of romidepsin and DAC increased the number of modulated genes involved in apoptosis and cell cycle arrest. Moreover, combinations of HDAC inhibitors and DNMT inhibitors such as romidepsin and azacytidine or DAC are also effective in the clinical setting [38, 39]. A clinical trial evaluating the combination of DAC, PDX, and pembrolizumab in PTCL is now underway (NCT03240211). We expect that the combination of PDX with DAC may be effective in relapsed/refractory PTCLs in the clinical setting.

## Conclusion

We established PDX-resistant T-cell acute lymphoblastic leukemia cell lines, and the combination of DAC and PDX exhibited a potent synergistic effect in these cells. Reduced cellular uptake of PDX and epigenetic alterations may contribute to the development PDX resistance. These findings will support clinical trials for patients with refractory/relapsed PTCL using PDX in combination with other agents.

## Supplementary Information

**Additional file 1: Supplementary Data 1.** Growth curves and doubling time. The growth curves of parental and PDX-resistant celllines are shown. The cells (1 × 10^5^ cells) were cultured for 96 h and counted every 12 hours. CEM/P, PDX-resistance CEM cell. MOLT4/P, PDX-resistance MOLT4 cell.

**Additional file 2: Supplementary Data 2.** DNA sequence of RFC1**.** created by author based on COSMIC v86. * Rothem L et al. *Biochem J. 2002;367,741–750. Zhao R* et al. *Mol Pharmacol.1999;56,68–76.* Sequencing analysis of exons 2 and 3 of *RFC1* (SLC19A1) was performed using genomic PCR and RT-PCR. (a, b) Distribution of point mutations in *RFC1*, as previously reported. (c) Sanger sequencing of RFC1 exon2 and exon3. There were no acquired somatic mutations in PDX-resistant cells. Exon 2 (MOLT4 and PDX-resistant cells), exon 3 (MOLT4 and PDX-resistant cells).

**Additional file 3: Supplementary Data 3.** IC_50_ values of other agents. Expression of folate metabolism pathway components. Total RNA (10 ng) from parental and PDX-resistant cells was obtained, and mRNA expression was evaluated using Clariom S Arrays. There were no significant differences in the expression levels in either PDX-resistant cell line. *SLC19A1*; solute carrier family 19 member 1(known as RFC1), *SLC46A1*; solute carrier family 46 member 1, *FPGS*; folylpolyglutamate synthase,* GGH;* gamma-glutamyl hydrolase, *DHFR*; dihydrofolate reductase, *TS*; thymidylate synthase. CEM/P, PDX-resistance CEM cell. MOLT4/P, PDX-resistance MOLT4 cell.

**Additional file 4: Supplementary Data 4.** The GESA results of CEM/P and MOLT4/P. Gene set enrichment analysis (GSEA). Significantly enriched gene signatures in GSEA analysis with microarray analysis from parental and PDX-resistant cells using the hallmark gene set. a) GSEA in CEM/P cell, b) GSEA in MOLT4/P cell.

**Additional file 5: Supplementary Data 5.** Comparison of PDX-resistant cells and DNMT3B knockdown cells. Viral supernatants containing DNMT3B-shRNA (TRCN0000035686) and the control non-target shRNA were purchased from Sigma-Aldrich. CEM/P cells were infected in the presence of 25 µg/mL retronectin (Takara Bio, Japan) for 6 h and selected with puromycin (10 mg/mL).PDX-resistant CEM cells were infected in the presence of 25 mg/mL of Retronectin for 6 hr and selected with puromycin (10 µg/mL). DNMT3B, DNA methyltransferase 3B.

## Data Availability

The datasets used and analyzed, together with the PDX-resistance cell line, during the current study are available from the corresponding author on reasonable request.

## Abbreviations

PDX: Pralatrexate
DHFR: Dihydrofolate reductase
PTCLs: Peripheral T-cell lymphomas
RFC1: Reduced folate carrier
FPGS: Folylpolyglutamate synthetase
MTX: Methotrexate
PROPEL: Patients with Relapsed or Refractory Peripheral T-Cell Lymphoma
CEM: CCRF-CEM
MOLT4: MOLT-4
FDS: Forodesine
AraC: Cytarabine
BOR: Bortezomib
DAC: Decitabine
dGuo: Deoxyguanosine
LBH589: Panobinostat
IC_50_: Inhibit 50% growth of the cells
XTT: 2,3-bis-(2-methoxy-4-nitro-5-sulfophenyl)-2 h-tetrazolium-5-carboxanilide
CI: Combination index
RT-qPCR: Reverse transcription-quantitative polymerase chain reaction
DNMT3B: DNA methyltransferase 3β
GGH: Gamma glutamyl hydrolase
HDAC: Histone deacetylase
GSEA: Gene set enrichment analysis
TSS: Transcription start site
TYMS: Thymidylate synthase
KDELC2: Known as POGLUT3, protein O-glucosyltransferase 3
CUL1: Cullin 1
EGLN1: Egl-9 family hypoxia inducible factor 1
CBFA2T3: CBFA2/RUNX1 partner transcriptional co-repressor 3
mTORC1: Mechanistic target of rapamycin complex1
