# Supplementary material for: Characterization of newly established Pralatrexate-resistant cell lines and the mechanisms of resistance
Source: BMC Cancer. 2021 Jul 31;21:879. doi: 10.1186/s12885-021-08607-9 (PMC8325835; doi:10.1186/s12885-021-08607-9)
Supplement: Supplementary file 1 — Additional file 1: Supplementary Data 1. Growth curves and doubling time. The growth curves of parental and PDX-resistant celllines are shown. The cells (1 × 10^5^ cells) were cultured for 96 h and counted every 12 hours. CEM/P, PDX-resistance CEM cell. MOLT4/P, PDX-resistance MOLT4 cell. [file 12885_2021_8607_MOESM1_ESM.docx]

**Supplementary Data 1. Growth curves and doubling time.**


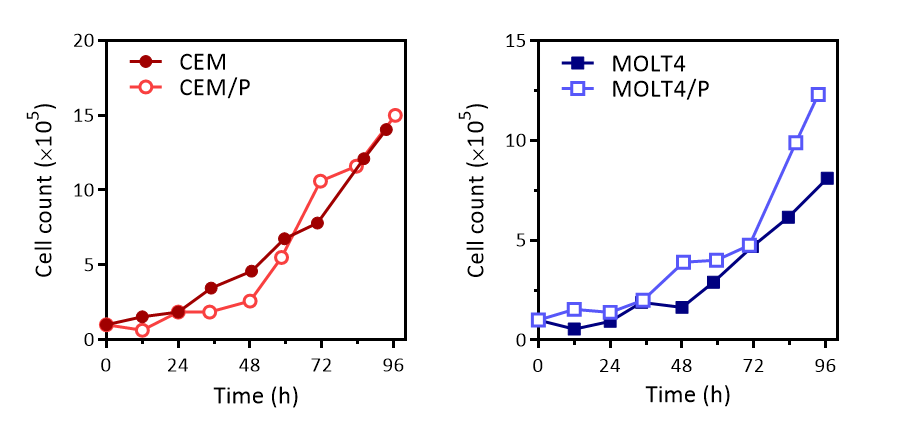


The growth curves of parental and PDX-resistant cell lines are shown. The cells (1 × 10^5^ cells) were cultured for 96 h and counted every 12 hours. CEM/P, PDX-resistance CEM cell. MOLT4/P, PDX-resistance MOLT4 cell.
