# Supplementary material for: Characterization of newly established Pralatrexate-resistant cell lines and the mechanisms of resistance
Source: BMC Cancer. 2021 Jul 31;21:879. doi: 10.1186/s12885-021-08607-9 (PMC8325835; doi:10.1186/s12885-021-08607-9)
Supplement: Supplementary file 2 — Additional file 2: Supplementary Data 2. DNA sequence of RFC1. created by author based on COSMIC v86. * Rothem L et al. Biochem J. 2002;367,741–750. Zhao R et al. Mol Pharmacol.1999;56,68–76. Sequencing analysis of exons 2 and 3 of RFC1 (SLC19A1) was performed using genomic PCR and RT-PCR. (a, b) Distribution of point mutations in RFC1, as previously reported. (c) Sanger sequencing of RFC1 exon2 and exon3. There were no acquired somatic mutations in PDX-resistant cells. Exon 2 (MOLT4 and PDX-resistant cells), exon 3 (MOLT4 and PDX-resistant cells). [file 12885_2021_8607_MOESM2_ESM.docx]

**Supplementary Data 2. DNA sequence of RFC1.**


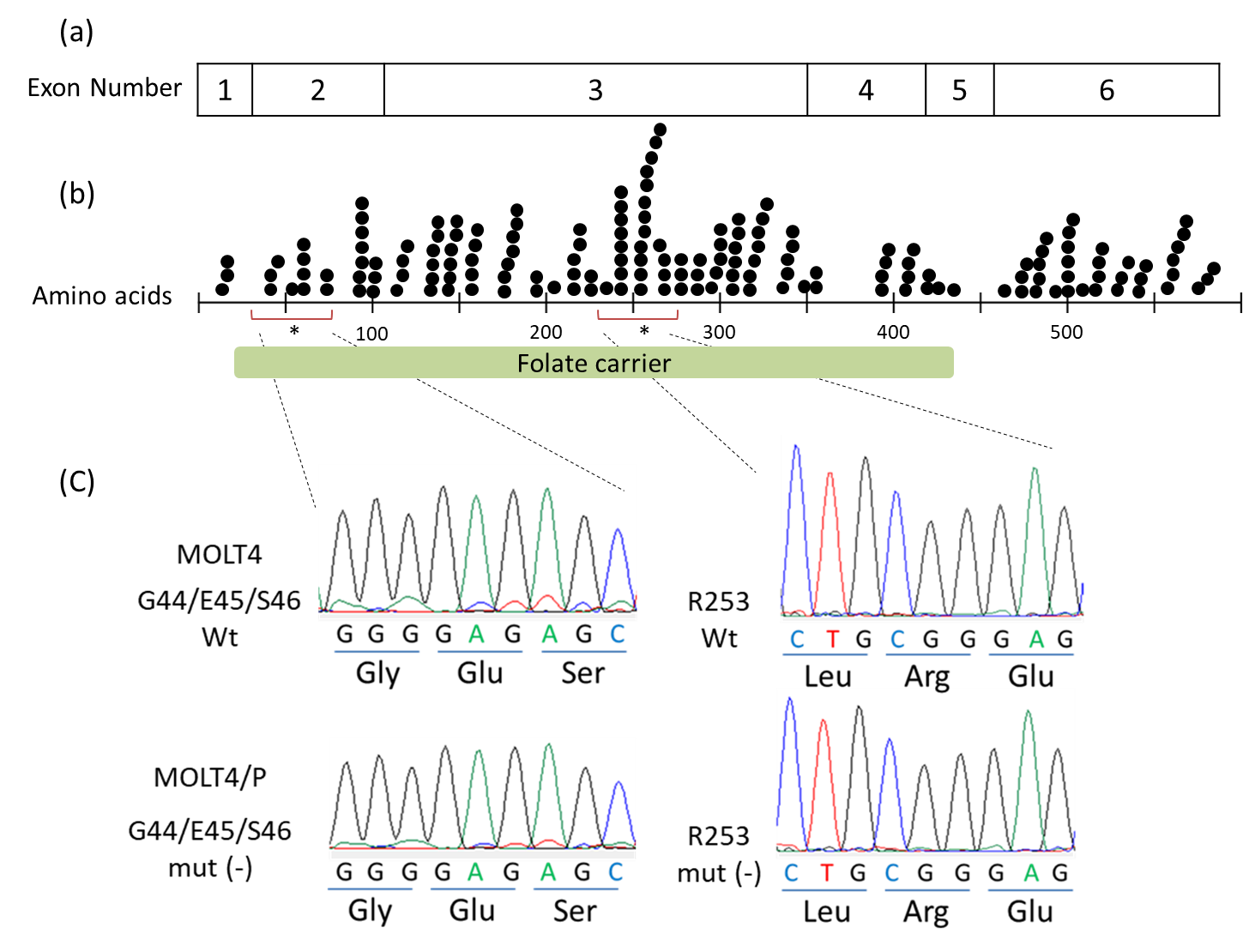


*created by author based on COSMIC v86.*

*** *Rothem L et al. Biochem J. 2002;367,741-750.*

*Zhao R et al. Mol Pharmacol.1999;56,68-76.*

Sequencing analysis of exons 2 and 3 of *RFC1* (SLC19A1) was performed using genomic PCR and RT-PCR. (a, b) Distribution of point mutations in *RFC1*, as previously reported. (c) Sanger sequencing of RFC1 exon2 and exon3. There were no acquired somatic mutations in PDX-resistant cells. Exon 2 (MOLT4 and PDX-resistant cells), exon 3 (MOLT4 and PDX-resistant cells).
