# Supplementary material for: Characterization of newly established Pralatrexate-resistant cell lines and the mechanisms of resistance
Source: BMC Cancer. 2021 Jul 31;21:879. doi: 10.1186/s12885-021-08607-9 (PMC8325835; doi:10.1186/s12885-021-08607-9)
Supplement: Supplementary file 3 — Additional file 3: Supplementary Data 3. IC50 values of other agents. Expression of folate metabolism pathway components. Total RNA (10 ng) from parental and PDX-resistant cells was obtained, and mRNA expression was evaluated using Clariom S Arrays. There were no significant differences in the expression levels in either PDX-resistant cell line. SLC19A1; solute carrier family 19 member 1(known as RFC1), SLC46A1; solute carrier family 46 member 1, FPGS; folylpolyglutamate synthase, GGH; gamma-glutamyl hydrolase, DHFR; dihydrofolate reductase, TS; thymidylate synthase. CEM/P, PDX-resistance CEM cell. MOLT4/P, PDX-resistance MOLT4 cell. [file 12885_2021_8607_MOESM3_ESM.docx]

**Supplementary Data 3. IC_50_ values of other agents.**

Expression of folate metabolism pathway components. Total RNA (10 ng) from parental and PDX-resistant cells was obtained, and mRNA expression was evaluated using Clariom S Arrays. There were no significant differences in the expression levels in either PDX-resistant cell line. *SLC19A1*; solute carrier family 19 member 1(known as RFC1), *SLC46A1*; solute carrier family 46 member 1, *FPGS*; folylpolyglutamate synthase, *GGH;* gamma-glutamyl hydrolase, *DHFR*; dihydrofolate reductase, *TS*; thymidylate synthase. CEM/P, PDX-resistance CEM cell. MOLT4/P, PDX-resistance MOLT4 cell.
