# Supplementary material for: Characterization of newly established Pralatrexate-resistant cell lines and the mechanisms of resistance
Source: BMC Cancer. 2021 Jul 31;21:879. doi: 10.1186/s12885-021-08607-9 (PMC8325835; doi:10.1186/s12885-021-08607-9)
Supplement: Supplementary file 4 — Additional file 4: Supplementary Data 4. The GESA results of CEM/P and MOLT4/P. Gene set enrichment analysis (GSEA). Significantly enriched gene signatures in GSEA analysis with microarray analysis from parental and PDX-resistant cells using the hallmark gene set. a) GSEA in CEM/P cell, b) GSEA in MOLT4/P cell. [file 12885_2021_8607_MOESM4_ESM.docx]

**Supplementary Data 4. The GESA results of CEM/P and MOLT4/P.**

a)


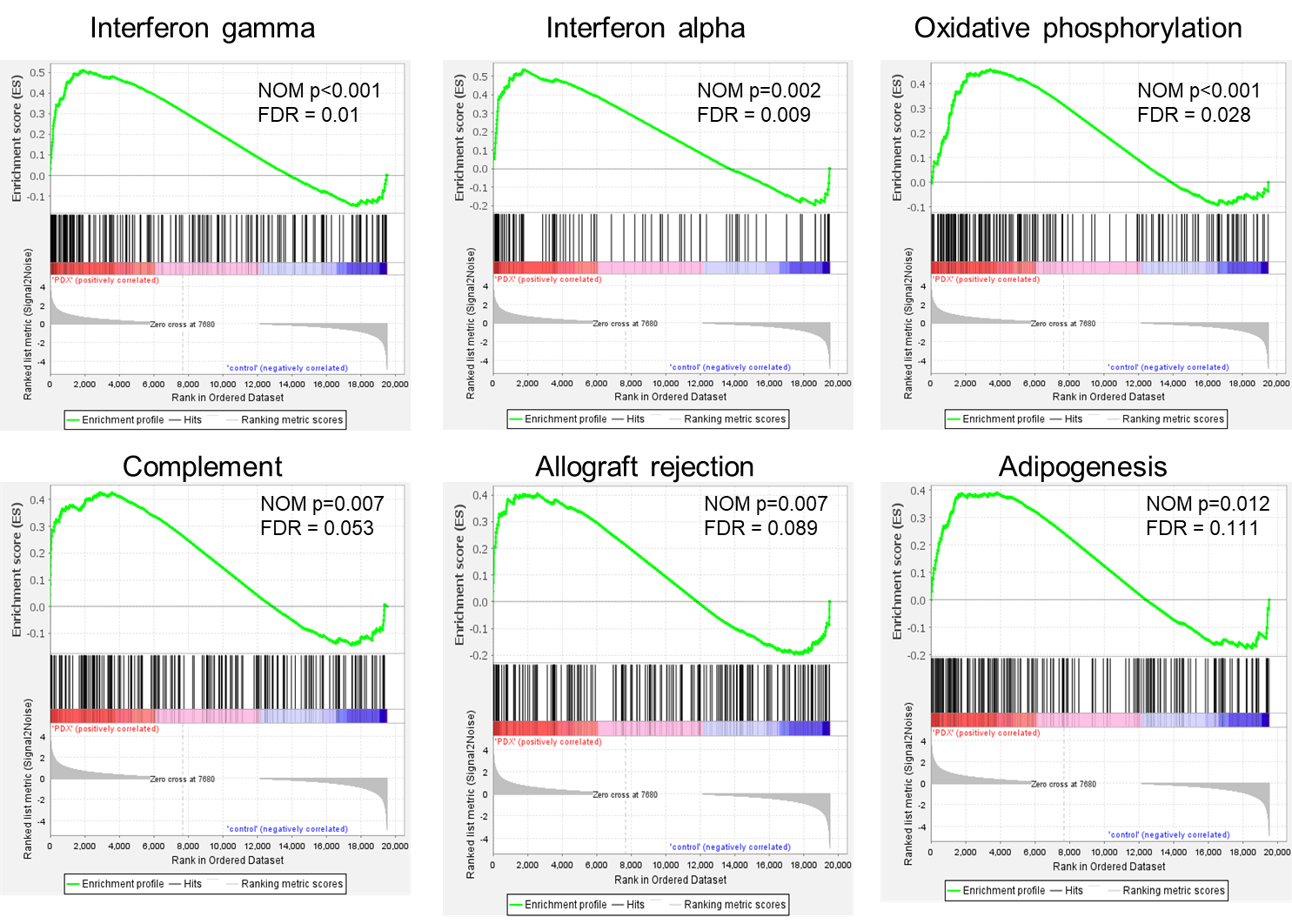


b)


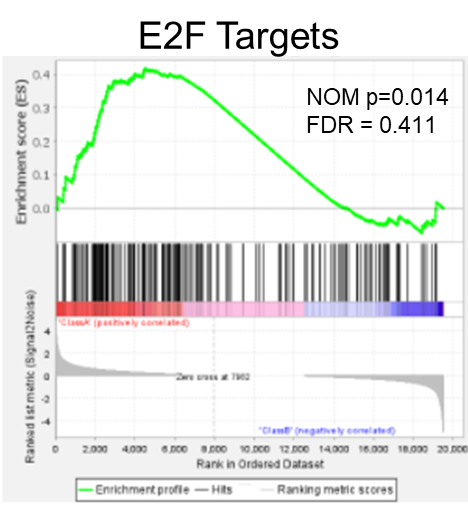


Gene set enrichment analysis (GSEA). Significantly enriched gene signatures in GSEA analysis with microarray analysis from parental and PDX-resistant cells using the hallmark gene set. a) GSEA in CEM/P cell, b) GSEA in MOLT4/P cell.
