# Supplementary material for: Characterization of newly established Pralatrexate-resistant cell lines and the mechanisms of resistance
Source: BMC Cancer. 2021 Jul 31;21:879. doi: 10.1186/s12885-021-08607-9 (PMC8325835; doi:10.1186/s12885-021-08607-9)
Supplement: Supplementary file 5 — Additional file 5: Supplementary Data 5. Comparison of PDX-resistant cells and DNMT3B knockdown cells. Viral supernatants containing DNMT3B-shRNA (TRCN0000035686) and the control non-target shRNA were purchased from Sigma-Aldrich. CEM/P cells were infected in the presence of 25 µg/mL retronectin (Takara Bio, Japan) for 6 h and selected with puromycin (10 mg/mL).PDX-resistant CEM cells were infected in the presence of 25 mg/mL of Retronectin for 6 hr and selected with puromycin (10 µg/mL). DNMT3B, DNA methyltransferase 3B. [file 12885_2021_8607_MOESM5_ESM.docx]

**Supplementary Data 5**

**Comparison of PDX-resistant cells and DNMT3B knockdown cells**

Viral supernatants containing DNMT3B-shRNA (TRCN0000035686) and the control non-target shRNA were purchased from Sigma-Aldrich. CEM/P cells were infected in the presence of 25 µg/mL retronectin (Takara Bio, Japan) for 6 h and selected with puromycin (10 mg/mL). PDX-resistant CEM cells were infected in the presence of 25 mg/mL of Retronectin for 6 hr and selected with puromycin (10 µg/mL). DNMT3B, DNA methyltransferase 3B.
